# Crystal structure of bis­[μ-(4-meth­oxy­phen­yl)methane­thiol­ato-κ^2^ *S*:*S*]bis­[chlorido­(η^6^-1-isopropyl-4-methyl­benzene)­ruthenium(II)] chloro­form disolvate

**DOI:** 10.1107/S2056989015017399

**Published:** 2015-09-26

**Authors:** David Stíbal, Georg Süss-Fink, Bruno Therrien

**Affiliations:** a Institut de Chimie, Université de Neuchâtel, Avenue de Bellevaux 51, CH-2000 Neuchâtel, Switzerland

**Keywords:** crystal structure, di­thiol­ato ruthenium(II) complex, *p*-cymene, hydrogen bonding

## Abstract

The mol­ecular structure of the title complex, [Ru_2_(C_8_H_9_OS)_2_Cl_2_(C_10_H_14_)_2_]·2CHCl_3_ or (*p*-MeC_6_H_4_Pr^*i*^)_2_Ru_2_(SCH_2_-*p*-C_6_H_5_-OCH_3_)_2_Cl_2_·2CHCl_3_, shows inversion symmetry. The two symmetry-related Ru^II^ atoms are bridged by two 4-meth­oxy-α-toluene­thiol­ato [(4-meth­oxy­phen­yl)methane­thiol­ato] units. One chlorido ligand and the *p*-cymene ligand complete the typical piano-stool coordination environment of the Ru^II^ atom. In the crystal, the CH moiety of the chloro­form mol­ecule inter­acts with the chlorido ligand of the dinuclear complex, while one Cl atom of the solvent inter­acts more weakly with the methyl group of the bridging 4-meth­oxy-α-toluene­thiol­ato unit. This assembly leads to the formation of supra­molecular chains extending parallel to [021].

## Abstract 2

In the title complex, the two symmetry-related Ru^II^ atoms are bridged by two 4-meth­oxy-α-toluene­thiol­ate [(4-meth­oxy­phen­yl)methane­thiol­ate] units. One chloride ligand and the *p*-cymene ligand complete the typical piano-stool coordination environment of the Ru^II^ atom. In the crystal, the CH moiety of the chloro­form mol­ecule inter­acts with the chloride ligand of the dinuclear complex, while one Cl atom of the solvent inter­acts more weakly with the methyl group of the bridging 4-meth­oxy-α-toluene­thiol­ate unit. This assembly leads to the formation of supra­molecular chains extending parallel to [021].

## Chemical context   

Several series of dinuclear tri­thiol­ato arene ruthenium(II) complexes have been synthesized by our group in recent years (Gras *et al.*, 2010 ▸; Giannini *et al.*, 2011 ▸, 2013*a* ▸) and investigated for their potential as anti­cancer agents (Giannini *et al.*, 2012 ▸). The *in vitro* studies showed the IC_50_ values of the chloride salts of these complexes to be regularly in the nanomolar range, being among the most active ruthenium complexes synthesized to date. The recent discovery of di­thiol­ato complexes (Ibao *et al.*, 2012 ▸) opened new possibilities for the design of thiol­ato-bridged dinuclear arene ruthenium(II) complexes (Giannini *et al.*, 2013*b* ▸). Herein we report the structure of a neutral di­thio­l­ato complex, *p*-MeC_6_H_4_Pr^*i*^)_2_Ru_2_(SCH_2_-*p*-C_6_H_5_-OCH_3_)_2_Cl_2_ that crystallized as a chloro­form disolvate.
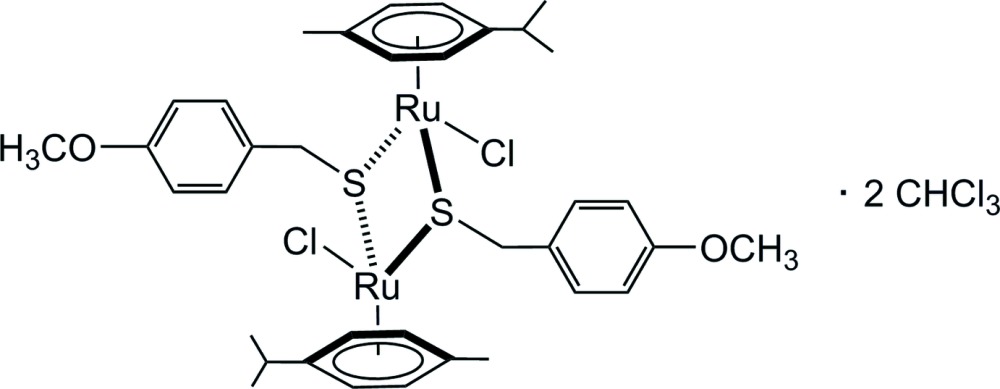


## Structural commentary   

**Figure 1:**
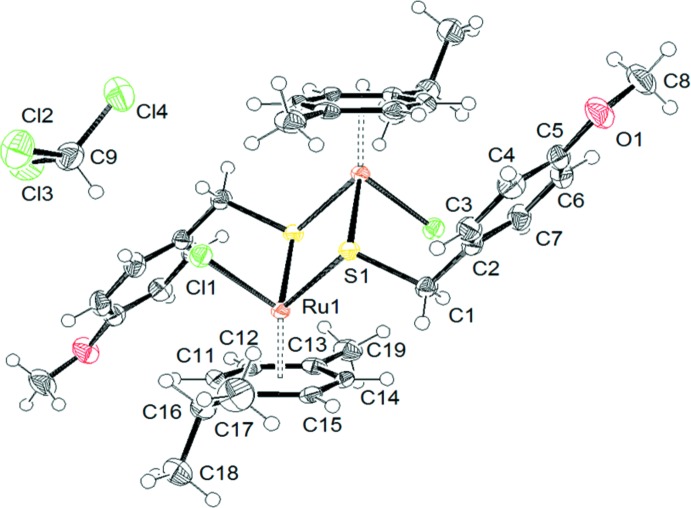
The mol­ecular structures of the components in the structure of (*p*-MeC_6_H_4_Pr^*i*^)_2_Ru_2_(SCH_2_-*p*-C_6_H_5_-OCH_3_)_2_Cl_2_·2CHCl_3_. Displacement ellipsoids are drawn at the 50% probability level.

The mol­ecular structure of the dinuclear title compound, [RuCl(C_8_H_9_OS)(C_10_H_14_)]_2_·2CHCl_3_, exhibits inversion symmetry and is presented in Fig. 1 ▸. The Ru^II^ atom adopts a typical piano-stool coordination geometry with the *p*-cymene ligand being bound facially, formally occupying three coord­ination sites. The other three positions are occupied by symmetry-related S atoms of two 4-meth­oxy-α-toluene­thiol­ato units and one chlorido ligand. The inter­atomic distances between Ru1 and the two symmetry-related S1 atoms are 2.3778 (10) and 2.3931 (10) Å, between Ru1 and Cl1 2.4284 (12) Å, and between S1 and C1 1.847 (3) Å. The Ru1—S1—Ru1^i^ angle is 100.03 (4)° [symmetry code: (i) –*x* + 1, –*y* + 1, –*z*]. The distance between the metal atom and the associated ring centroid (C1–C6) is 1.684 Å. In agreement with the electronic count, there is no metal–metal bond, the Ru⋯Ru distance in the dinuclear complex mol­ecule being 3.6555 (9) Å.

## Supra­molecular features   

**Figure 2:**
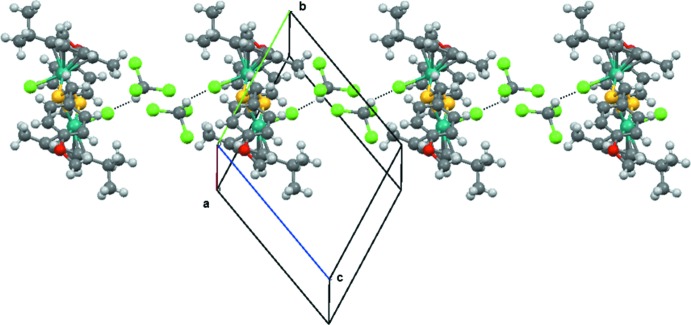
The one dimensional supra­molecular network in the crystal packing of (*p*-MeC_6_H_4_Pr^*i*^)_2_Ru_2_(SCH_2_-*p*-C_6_H_5_-OCH_3_)_2_Cl_2_·2CHCl_3_. Only the stronger of the C—H⋯Cl inter­actions is shown (dotted lines).

In the crystal packing of the title compound, the chlorido ligand of the complex inter­acts with the CH moiety of the chloro­form mol­ecule. Moreover, a more weak hydrogen-bonding inter­action is also observed between the meth­oxy group of the 4-meth­oxy-α-toluene­thiol­ato and a chlorine atom of the solvent mol­ecule (Table 1 ▸). These inter­actions give rise to the formation of supra­molecular chains extending parallel to [021] (Fig. 2 ▸).

## Synthesis and crystallization   

The title complex was obtained from the reaction of 100 mg (0.163 mmol) of (*p*-MeC_6_H_4_Pr^*i*^)_2_Ru_2_Cl_4_ and 50.3 µl (0.343 mmol) of 4-meth­oxy-α-toluene­thiol in ethanol. The solution was stirred at room temperature for 3 h, afterwards the solvent was reduced to 2 ml *in vacuo* and the product precipitated by adding hexane. The solid was filtered, washed with hexane and dried *in vacuo*. X-ray quality crystals were obtained by slow diffusion of diethyl ether into the solution of the title complex in chloro­form.

Yield: 124.2 mg (89%). C_36_H_46_Cl_2_O_2_Ru_2_S_2_: calculated C, 50.99; H, 5.47; found C, 50.76; H, 5.46. ESI MS: (MeOH + CH_2_Cl_2_): *m*/*z* = 822.8 [M − Cl]^+. 1^H NMR (400 MHz, CDCl_3_): δ = 7.49 (*d*, ^3^ *J* = 8 Hz, 2H, SCH_2_C_6_H_4_-*p*-OCH_3_), 6.85 (*d*, ^3^ *J* = 8 Hz, 2H, SCH_2_C_6_H_4_-*p*-OCH_3_), 5.15–4.89 [*m*, 8H, *p*-CH_3_C_6_ **H**_4_CH(CH_3_)_2_], 4.15 (*d*, ^3^ *J* = 11 Hz, 2H, SCH_2_C_6_ **H**_4_-*p*-OCH_3_), 3.83 (*s*, 6H, SCH_2_C_6_H_4_-*p*-OC**H**_3_), 3.26 (*d*, ^3^ *J* = 11 Hz, 2H, SC**H**_2_C_6_H_4_-*p*-OCH_3_), 2.86 [sept, ^3^ *J* = 8 Hz, 2H, *p*-CH_3_C_6_H_4_C**H**(CH_3_)_2_], 1.89 [*s*, 6H, *p*-C**H**_3_C_6_H_4_CH(CH_3_)_2_], 1.2 [*s*, 12H, *p*-CH_3_C_6_H_4_CH(C**H**_3_)_2_] p.p.m. ^13^C NMR (100 MHz, CDCl_3_): δ = 158.41, 132.89, 131.56, 112.96, 96.97, 83.91, 83.03, 55.32, 35.90, 29.88, 23.50, 21.22, 18.79 p.p.m.

## Refinement   

Crystal data, data collection and structure refinement details are summarized in Table 2 ▸. All hydrogen atoms were included in calculated positions and treated as riding atoms, with C—H = 0.93 Å for C_aromatic_, 0.97 Å for –CH_2_–, 0.98 Å for –CH–, and 0.96 Å for –CH_3_, with *U*_iso_(H) = 1.2*U*_eq_(C) and 1.5*U*_eq_(C) for methyl H atoms.

## Supplementary Material

Crystal structure: contains datablock(s) I, global. DOI: 10.1107/S2056989015017399/wm5217sup1.cif

Structure factors: contains datablock(s) I. DOI: 10.1107/S2056989015017399/wm5217Isup2.hkl

CCDC reference: 1415346

Additional supporting information:  crystallographic information; 3D view; checkCIF report

## Figures and Tables

**Table 1:** Hydrogen-bond geometry (, )

*D*H*A*	*D*H	H*A*	*D* *A*	*D*H*A*
C9H9Cl1	0.98	2.66	3.583(4)	157
C8H8*C*Cl4^i^	0.96	3.03	3.886(5)	150

Symmetry code: (i) 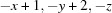.

**Table 2:** Experimental details

Crystal data	
Chemical formula	[Ru_2_(C_8_H_9_OS)_2_Cl_2_(C_10_H_14_)_2_]2CHCl_3_
*M*_r_	1086.62
Crystal system, space group	Triclinic, *P* 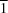
Temperature (K)	173
*a*, *b*, *c* ()	10.034(2), 10.070(2), 12.124(2)
, , ()	112.75(3), 95.58(3), 98.51(3)
*V* (^3^)	1101.2(4)
*Z*	1
Radiation type	Mo *K*
(mm^1^)	1.30
Crystal size (mm)	0.24 0.21 0.19

Data collection	
Diffractometer	STOE IPDS
Absorption correction	Empirical (using intensity measurements) (Walker Stuart, 1983 ▸)
*T*_min_, *T*_max_	0.655, 0.819
No. of measured, independent and observed [*I* > 2(*I*)] reflections	13000, 5787, 4504
*R*_int_	0.058
(sin /)_max_ (^1^)	0.689

Refinement	
*R*[*F* ^2^ > 2(*F* ^2^)], *wR*(*F* ^2^), *S*	0.039, 0.092, 0.97
No. of reflections	5787
No. of parameters	239
H-atom treatment	H-atom parameters constrained
_max_, _min_ (e ^3^)	0.94, 1.47

Computer programs: *EXPOSE*, *CELL* and *INTEGRATE* (*IPDS Software*; Stoe Cie, 2000 ▸), *SHELXS97* (Sheldrick, 2008 ▸), *SHELXL2014*/7 (Sheldrick, 2015 ▸) and *ORTEP-3 for Windows* (Farrugia, 2012 ▸).

## supplementary crystallographic information

### Crystal data

**Table:**

[Ru_2_(C_8_H_9_OS)_2_Cl_2_(C_10_H_14_)_2_]·2CHCl_3_	*Z* = 1
*M**_r_* = 1086.62	*F*(000) = 548
Triclinic, *P*1	*D*_x_ = 1.639 Mg m^−^^3^
Hall symbol: -P 1	Mo *K*α radiation, λ = 0.71073 Å
*a* = 10.034 (2) Å	Cell parameters from 8000 reflections
*b* = 10.070 (2) Å	θ = 2.1–28.9°
*c* = 12.124 (2) Å	µ = 1.30 mm^−^^1^
α = 112.75 (3)°	*T* = 173 K
β = 95.58 (3)°	Block, orange
γ = 98.51 (3)°	0.24 × 0.21 × 0.19 mm
*V* = 1101.2 (4) Å^3^	

### Data collection

**Table:**

STOE IPDS diffractometer	5787 independent reflections
Radiation source: fine-focus sealed tube	4504 reflections with *I* > 2σ(*I*)
Detector resolution: 0.81 pixels mm^-1^	*R*_int_ = 0.058
phi oscillation scans	θ_max_ = 29.3°, θ_min_ = 1.9°
Absorption correction: empirical (using intensity measurements) (Walker & Stuart, 1983)	*h* = −11→13
*T*_min_ = 0.655, *T*_max_ = 0.819	*k* = −13→13
13000 measured reflections	*l* = −16→16

### Refinement

**Table:**

Refinement on *F*^2^	0 restraints
Least-squares matrix: full	Hydrogen site location: inferred from neighbouring sites
*R*[*F*^2^ > 2σ(*F*^2^)] = 0.039	H-atom parameters constrained
*wR*(*F*^2^) = 0.092	*w* = 1/[σ^2^(*F*_o_^2^) + (0.047*P*)^2^] where *P* = (*F*_o_^2^ + 2*F*_c_^2^)/3
*S* = 0.97	(Δ/σ)_max_ < 0.001
5787 reflections	Δρ_max_ = 0.94 e Å^−^^3^
239 parameters	Δρ_min_ = −1.47 e Å^−^^3^

### Special details

**Table:**

Experimental. A crystal was mounted at 173 K on a Stoe Image Plate Diffraction System (Stoe & Cie, 2000) using Mo *K*α graphite monochromated radiation. Image plate distance 100 mm, φ oscillation scans 0 - 180°, step Δφ = 1.2°, 5 minutes per frame.Reflections were merged by *SHELXL* according to the crystal class for the calculation of statistics and refinement._reflns_Friedel_fraction is defined as the number of unique Friedel pairs measured divided by the number that would be possible theoretically, ignoring centric projections and systematic absences.
Geometry. All e.s.d.'s (except the e.s.d. in the dihedral angle between two l.s. planes) are estimated using the full covariance matrix. The cell e.s.d.'s are taken into account individually in the estimation of e.s.d.'s in distances, angles and torsion angles; correlations between e.s.d.'s in cell parameters are only used when they are defined by crystal symmetry. An approximate (isotropic) treatment of cell e.s.d.'s is used for estimating e.s.d.'s involving l.s. planes.
Refinement. Refinement of *F*^2^ against ALL reflections. The weighted *R*-factor *wR* and goodness of fit *S* are based on *F*^2^, conventional *R*-factors *R* are based on *F*, with *F* set to zero for negative *F*^2^. The threshold expression of *F*^2^ > σ(*F*^2^) is used only for calculating *R*-factors(gt) *etc*. and is not relevant to the choice of reflections for refinement. *R*-factors based on *F*^2^ are statistically about twice as large as those based on *F*, and *R*- factors based on ALL data will be even larger.

### Fractional atomic coordinates and isotropic or equivalent isotropic displacement parameters (Å^2^)

**Table:**

	*x*	*y*	*z*	*U*_iso_*/*U*_eq_	
C1	0.7926 (3)	0.4925 (4)	−0.0177 (3)	0.0255 (6)	
H1A	0.8508	0.4762	0.0426	0.031*	
H1B	0.7565	0.3986	−0.0855	0.031*	
C2	0.8739 (3)	0.5957 (4)	−0.0603 (3)	0.0247 (6)	
C3	0.9603 (3)	0.7265 (4)	0.0208 (3)	0.0282 (7)	
H3	0.9717	0.7483	0.1035	0.034*	
C4	1.0290 (3)	0.8237 (4)	−0.0184 (3)	0.0314 (7)	
H4	1.0850	0.9107	0.0376	0.038*	
C5	1.0151 (3)	0.7924 (4)	−0.1422 (3)	0.0297 (7)	
C6	0.9324 (3)	0.6622 (4)	−0.2252 (3)	0.0326 (7)	
H6	0.9230	0.6397	−0.3079	0.039*	
C7	0.8633 (3)	0.5650 (4)	−0.1833 (3)	0.0303 (7)	
H17	0.8086	0.4771	−0.2393	0.036*	
C8	1.0626 (4)	0.8763 (5)	−0.2960 (4)	0.0471 (10)	
H8A	1.0848	0.7849	−0.3464	0.071*	
H8B	1.1185	0.9558	−0.3056	0.071*	
H8C	0.9679	0.8746	−0.3195	0.071*	
C9	0.2694 (4)	0.9102 (5)	0.3930 (4)	0.0423 (9)	
H9	0.3270	0.8367	0.3788	0.051*	
C10	0.5851 (3)	0.3518 (4)	0.2545 (3)	0.0260 (7)	
C11	0.4405 (4)	0.2937 (4)	0.2169 (3)	0.0294 (7)	
H11	0.3833	0.3152	0.2747	0.035*	
C12	0.3845 (3)	0.2066 (4)	0.0967 (3)	0.0273 (7)	
H12	0.2906	0.1703	0.0758	0.033*	
C13	0.4679 (3)	0.1709 (3)	0.0033 (3)	0.0242 (6)	
C14	0.6104 (3)	0.2285 (4)	0.0405 (3)	0.0247 (6)	
H14	0.6674	0.2094	−0.0175	0.030*	
C15	0.6681 (3)	0.3150 (4)	0.1645 (3)	0.0243 (6)	
H15	0.7624	0.3478	0.1864	0.029*	
C16	0.6405 (4)	0.4500 (5)	0.3870 (3)	0.0371 (8)	
H16	0.5723	0.5092	0.4176	0.044*	
C17	0.7731 (5)	0.5567 (5)	0.4064 (4)	0.0516 (11)	
H17A	0.8449	0.5032	0.3844	0.077*	
H17B	0.7962	0.6241	0.4902	0.077*	
H17C	0.7621	0.6105	0.3569	0.077*	
C18	0.6510 (5)	0.3546 (6)	0.4589 (4)	0.0528 (12)	
H18A	0.5627	0.2956	0.4491	0.079*	
H18B	0.6824	0.4168	0.5433	0.079*	
H18C	0.7144	0.2918	0.4293	0.079*	
C19	0.4071 (4)	0.0830 (4)	−0.1272 (3)	0.0360 (8)	
H19A	0.4595	0.1167	−0.1768	0.054*	
H19B	0.3145	0.0947	−0.1409	0.054*	
H19C	0.4081	−0.0189	−0.1477	0.054*	
O1	1.0868 (3)	0.8961 (3)	−0.1727 (3)	0.0419 (6)	
S1	0.65082 (7)	0.57453 (8)	0.04876 (7)	0.02020 (15)	
Cl1	0.46757 (8)	0.64213 (9)	0.25227 (7)	0.02648 (16)	
Cl2	0.33078 (13)	1.05269 (14)	0.53676 (10)	0.0565 (3)	
Cl3	0.09992 (13)	0.82576 (14)	0.38522 (11)	0.0576 (3)	
Cl4	0.27665 (14)	0.97961 (17)	0.28011 (11)	0.0623 (3)	
Ru1	0.50634 (2)	0.41162 (3)	0.10619 (2)	0.01824 (7)	

### Atomic displacement parameters (Å^2^)

**Table:**

	*U*^11^	*U*^22^	*U*^33^	*U*^12^	*U*^13^	*U*^23^
C1	0.0191 (14)	0.0273 (18)	0.0344 (17)	0.0071 (12)	0.0081 (12)	0.0157 (15)
C2	0.0176 (13)	0.0288 (18)	0.0326 (16)	0.0072 (12)	0.0081 (12)	0.0157 (14)
C3	0.0246 (15)	0.0313 (19)	0.0302 (16)	0.0052 (13)	0.0046 (13)	0.0140 (15)
C4	0.0245 (16)	0.0288 (19)	0.0375 (18)	0.0003 (13)	0.0042 (14)	0.0120 (16)
C5	0.0234 (15)	0.0292 (19)	0.0413 (19)	0.0046 (13)	0.0132 (14)	0.0180 (16)
C6	0.0268 (16)	0.042 (2)	0.0302 (17)	0.0061 (15)	0.0091 (13)	0.0154 (16)
C7	0.0265 (16)	0.0290 (19)	0.0327 (17)	0.0021 (13)	0.0087 (14)	0.0100 (15)
C8	0.045 (2)	0.057 (3)	0.054 (2)	0.0045 (19)	0.0187 (19)	0.038 (2)
C9	0.048 (2)	0.040 (2)	0.037 (2)	0.0147 (19)	0.0076 (18)	0.0118 (18)
C10	0.0295 (16)	0.0318 (18)	0.0266 (15)	0.0132 (13)	0.0079 (13)	0.0191 (15)
C11	0.0337 (17)	0.0343 (19)	0.0338 (17)	0.0137 (14)	0.0159 (14)	0.0236 (16)
C12	0.0243 (15)	0.0214 (17)	0.0428 (19)	0.0030 (12)	0.0072 (13)	0.0202 (15)
C13	0.0315 (16)	0.0122 (14)	0.0285 (15)	0.0005 (12)	0.0010 (13)	0.0101 (13)
C14	0.0282 (15)	0.0224 (16)	0.0331 (16)	0.0141 (13)	0.0139 (13)	0.0162 (14)
C15	0.0236 (14)	0.0250 (17)	0.0311 (16)	0.0118 (12)	0.0055 (12)	0.0160 (14)
C16	0.044 (2)	0.047 (2)	0.0249 (16)	0.0214 (18)	0.0061 (15)	0.0161 (17)
C17	0.056 (3)	0.050 (3)	0.034 (2)	0.009 (2)	−0.0070 (19)	0.006 (2)
C18	0.063 (3)	0.079 (4)	0.036 (2)	0.034 (3)	0.015 (2)	0.035 (2)
C19	0.046 (2)	0.0240 (19)	0.0357 (19)	0.0037 (15)	0.0020 (16)	0.0123 (16)
O1	0.0349 (14)	0.0449 (17)	0.0514 (16)	−0.0023 (12)	0.0138 (12)	0.0275 (14)
S1	0.0175 (3)	0.0209 (4)	0.0245 (4)	0.0040 (3)	0.0043 (3)	0.0116 (3)
Cl1	0.0281 (4)	0.0250 (4)	0.0260 (4)	0.0070 (3)	0.0072 (3)	0.0088 (3)
Cl2	0.0583 (7)	0.0529 (7)	0.0386 (5)	0.0077 (5)	0.0022 (5)	0.0004 (5)
Cl3	0.0537 (6)	0.0547 (7)	0.0578 (7)	0.0009 (5)	0.0024 (5)	0.0211 (6)
Cl4	0.0711 (8)	0.0793 (9)	0.0511 (6)	0.0268 (7)	0.0240 (6)	0.0348 (7)
Ru1	0.01758 (11)	0.01881 (13)	0.02142 (12)	0.00467 (8)	0.00493 (8)	0.01074 (10)

### Geometric parameters (Å, º)

**Table:**

C1—C2	1.503 (4)	C11—H11	0.9300
C1—S1	1.847 (3)	C12—C13	1.439 (5)
C1—H1A	0.9700	C12—Ru1	2.194 (3)
C1—H1B	0.9700	C12—H12	0.9300
C2—C7	1.391 (5)	C13—C14	1.418 (5)
C2—C3	1.396 (5)	C13—C19	1.492 (5)
C3—C4	1.372 (5)	C13—Ru1	2.208 (3)
C3—H3	0.9300	C14—C15	1.422 (5)
C4—C5	1.397 (5)	C14—Ru1	2.173 (3)
C4—H4	0.9300	C14—H14	0.9300
C5—O1	1.369 (4)	C15—Ru1	2.204 (3)
C5—C6	1.384 (5)	C15—H15	0.9300
C6—C7	1.396 (5)	C16—C17	1.517 (6)
C6—H6	0.9300	C16—C18	1.534 (5)
C7—H17	0.9300	C16—H16	0.9800
C8—O1	1.421 (5)	C17—H17A	0.9600
C8—H8A	0.9600	C17—H17B	0.9600
C8—H8B	0.9600	C17—H17C	0.9600
C8—H8C	0.9600	C18—H18A	0.9600
C9—Cl2	1.752 (4)	C18—H18B	0.9600
C9—Cl3	1.761 (5)	C18—H18C	0.9600
C9—Cl4	1.764 (4)	C19—H19A	0.9600
C9—H9	0.9800	C19—H19B	0.9600
C10—C15	1.406 (4)	C19—H19C	0.9600
C10—C11	1.438 (5)	S1—Ru1	2.3778 (10)
C10—C16	1.517 (5)	S1—Ru1^i^	2.3931 (10)
C10—Ru1	2.219 (3)	Cl1—Ru1	2.4284 (12)
C11—C12	1.384 (5)	Ru1—S1^i^	2.3931 (10)
C11—Ru1	2.196 (3)		
			
C2—C1—S1	108.8 (2)	C14—C15—Ru1	69.85 (16)
C2—C1—H1A	109.9	C10—C15—H15	119.4
S1—C1—H1A	109.9	C14—C15—H15	119.4
C2—C1—H1B	109.9	Ru1—C15—H15	131.6
S1—C1—H1B	109.9	C17—C16—C10	113.6 (3)
H1A—C1—H1B	108.3	C17—C16—C18	112.9 (4)
C7—C2—C3	117.4 (3)	C10—C16—C18	109.4 (4)
C7—C2—C1	120.7 (3)	C17—C16—H16	106.9
C3—C2—C1	121.9 (3)	C10—C16—H16	106.9
C4—C3—C2	121.6 (3)	C18—C16—H16	106.9
C4—C3—H3	119.2	C16—C17—H17A	109.5
C2—C3—H3	119.2	C16—C17—H17B	109.5
C3—C4—C5	120.3 (3)	H17A—C17—H17B	109.5
C3—C4—H4	119.9	C16—C17—H17C	109.5
C5—C4—H4	119.9	H17A—C17—H17C	109.5
O1—C5—C6	124.3 (3)	H17B—C17—H17C	109.5
O1—C5—C4	116.1 (3)	C16—C18—H18A	109.5
C6—C5—C4	119.6 (3)	C16—C18—H18B	109.5
C5—C6—C7	119.2 (3)	H18A—C18—H18B	109.5
C5—C6—H6	120.4	C16—C18—H18C	109.5
C7—C6—H6	120.4	H18A—C18—H18C	109.5
C2—C7—C6	121.9 (3)	H18B—C18—H18C	109.5
C2—C7—H17	119.0	C13—C19—H19A	109.5
C6—C7—H17	119.0	C13—C19—H19B	109.5
O1—C8—H8A	109.5	H19A—C19—H19B	109.5
O1—C8—H8B	109.5	C13—C19—H19C	109.5
H8A—C8—H8B	109.5	H19A—C19—H19C	109.5
O1—C8—H8C	109.5	H19B—C19—H19C	109.5
H8A—C8—H8C	109.5	C5—O1—C8	117.6 (3)
H8B—C8—H8C	109.5	C1—S1—Ru1	111.36 (10)
Cl2—C9—Cl3	110.4 (2)	C1—S1—Ru1^i^	109.65 (11)
Cl2—C9—Cl4	110.0 (2)	Ru1—S1—Ru1^i^	100.03 (4)
Cl3—C9—Cl4	109.8 (2)	C14—Ru1—C12	67.73 (12)
Cl2—C9—H9	108.8	C14—Ru1—C11	79.89 (12)
Cl3—C9—H9	108.8	C12—Ru1—C11	36.75 (14)
Cl4—C9—H9	108.8	C14—Ru1—C15	37.92 (12)
C15—C10—C11	117.4 (3)	C12—Ru1—C15	79.44 (12)
C15—C10—C16	123.3 (3)	C11—Ru1—C15	67.08 (12)
C11—C10—C16	119.3 (3)	C14—Ru1—C13	37.76 (12)
C15—C10—Ru1	70.89 (16)	C12—Ru1—C13	38.17 (12)
C11—C10—Ru1	70.12 (16)	C11—Ru1—C13	68.07 (13)
C16—C10—Ru1	129.3 (2)	C15—Ru1—C13	68.35 (13)
C12—C11—C10	121.5 (3)	C14—Ru1—C10	68.23 (12)
C12—C11—Ru1	71.54 (17)	C12—Ru1—C10	67.85 (13)
C10—C11—Ru1	71.86 (17)	C11—Ru1—C10	38.02 (13)
C12—C11—H11	119.2	C15—Ru1—C10	37.08 (11)
C10—C11—H11	119.2	C13—Ru1—C10	81.53 (13)
Ru1—C11—H11	130.0	C14—Ru1—S1	96.92 (8)
C11—C12—C13	121.6 (3)	C12—Ru1—S1	159.60 (10)
C11—C12—Ru1	71.71 (19)	C11—Ru1—S1	157.71 (10)
C13—C12—Ru1	71.44 (17)	C15—Ru1—S1	96.80 (8)
C11—C12—H12	119.2	C13—Ru1—S1	121.79 (9)
C13—C12—H12	119.2	C10—Ru1—S1	120.29 (9)
Ru1—C12—H12	130.4	C14—Ru1—S1^i^	113.67 (9)
C14—C13—C12	116.8 (3)	C12—Ru1—S1^i^	93.66 (9)
C14—C13—C19	121.5 (3)	C11—Ru1—S1^i^	121.67 (10)
C12—C13—C19	121.7 (3)	C15—Ru1—S1^i^	151.25 (9)
C14—C13—Ru1	69.78 (18)	C13—Ru1—S1^i^	88.92 (9)
C12—C13—Ru1	70.38 (18)	C10—Ru1—S1^i^	159.68 (9)
C19—C13—Ru1	128.3 (2)	S1—Ru1—S1^i^	79.97 (4)
C13—C14—C15	121.5 (3)	C14—Ru1—Cl1	155.31 (10)
C13—C14—Ru1	72.46 (17)	C12—Ru1—Cl1	117.94 (9)
C15—C14—Ru1	72.23 (17)	C11—Ru1—Cl1	92.05 (10)
C13—C14—H14	119.3	C15—Ru1—Cl1	117.51 (10)
C15—C14—H14	119.3	C13—Ru1—Cl1	155.93 (9)
Ru1—C14—H14	128.4	C10—Ru1—Cl1	91.04 (9)
C10—C15—C14	121.1 (3)	S1—Ru1—Cl1	81.71 (4)
C10—C15—Ru1	72.04 (17)	S1^i^—Ru1—Cl1	90.48 (4)
			
S1—C1—C2—C7	−105.6 (3)	C11—C12—C13—Ru1	−53.5 (3)
S1—C1—C2—C3	73.2 (3)	C12—C13—C14—C15	1.2 (4)
C7—C2—C3—C4	2.1 (5)	C19—C13—C14—C15	178.6 (3)
C1—C2—C3—C4	−176.7 (3)	Ru1—C13—C14—C15	55.3 (2)
C2—C3—C4—C5	−0.9 (5)	C12—C13—C14—Ru1	−54.1 (2)
C3—C4—C5—O1	179.6 (3)	C19—C13—C14—Ru1	123.3 (3)
C3—C4—C5—C6	−0.5 (5)	C11—C10—C15—C14	2.4 (4)
O1—C5—C6—C7	−179.6 (3)	C16—C10—C15—C14	−176.8 (3)
C4—C5—C6—C7	0.6 (5)	Ru1—C10—C15—C14	−51.8 (3)
C3—C2—C7—C6	−2.0 (5)	C11—C10—C15—Ru1	54.2 (2)
C1—C2—C7—C6	176.8 (3)	C16—C10—C15—Ru1	−125.0 (3)
C5—C6—C7—C2	0.7 (5)	C13—C14—C15—C10	−2.7 (4)
C15—C10—C11—C12	−0.8 (4)	Ru1—C14—C15—C10	52.8 (3)
C16—C10—C11—C12	178.4 (3)	C13—C14—C15—Ru1	−55.4 (2)
Ru1—C10—C11—C12	53.7 (3)	C15—C10—C16—C17	25.9 (5)
C15—C10—C11—Ru1	−54.6 (2)	C11—C10—C16—C17	−153.3 (3)
C16—C10—C11—Ru1	124.7 (3)	Ru1—C10—C16—C17	−65.9 (4)
C10—C11—C12—C13	−0.5 (5)	C15—C10—C16—C18	−101.2 (4)
Ru1—C11—C12—C13	53.3 (3)	C11—C10—C16—C18	79.6 (4)
C10—C11—C12—Ru1	−53.9 (3)	Ru1—C10—C16—C18	167.0 (3)
C11—C12—C13—C14	0.3 (4)	C6—C5—O1—C8	7.4 (5)
Ru1—C12—C13—C14	53.8 (2)	C4—C5—O1—C8	−172.7 (3)
C11—C12—C13—C19	−177.0 (3)	C2—C1—S1—Ru1	175.94 (19)
Ru1—C12—C13—C19	−123.6 (3)	C2—C1—S1—Ru1^i^	66.2 (2)

Symmetry code: (i) −*x*+1, −*y*+1, −*z*.

### Hydrogen-bond geometry (Å, º)

**Table:**

*D*—H···*A*	*D*—H	H···*A*	*D*···*A*	*D*—H···*A*
C9—H9···Cl1	0.98	2.66	3.583 (4)	157
C8—H8*C*···Cl4^ii^	0.96	3.03	3.886 (5)	150

Symmetry code: (ii) −*x*+1, −*y*+2, −*z*.
